# Bidirectional referral system between National Taiwan University Hospital medical center and Zhongxiao Branch community hospital of Taipei City Hospital: focus on patient satisfaction

**DOI:** 10.1038/s41598-023-39794-z

**Published:** 2023-09-07

**Authors:** Mu-Jung Kao, Jenn Yeu Wang, Hsiao-Yun Hu

**Affiliations:** 1 Division of Nephrology and Division of Occupational Medicine, Department of Medicine, Taipei City Hospital, Zhongxiao Branch, No.87, Tongde Rd., Nangang Dist., Taipei City, 115006 Taiwan; 2 University of Taipei, Taipei City, Taiwan; 3 China Medical University, Taichung, Taiwan; 4 Taipei Medical University, Taipei City, Taiwan; 5 School of Medicine, Fu Jen Catholic University, Taipei City, Taiwan; 6 School of Medicine, National Yang-Ming Chiao Tung University, Hsinchu, Taiwan; 7 Department of Education and Research, Taipei City Hospital, Taipei City, Taiwan

**Keywords:** Health care, Medical research

## Abstract

The policy of bidirectional referral between National Taiwan University Hospital and Taipei City Hospital has been launched due to the over-crowding of the emergency department at National Taiwan University Hospital. This research aims to evaluate patient satisfaction with the bidirectional referral. Sixty-six patients have been referred from the emergency department of National Taiwan University Hospital to Taipei City Hospital, Zhongxiao campus from April 2015 to December 2017. The selection criteria of the subjects for bidirectional referral include the management of patients classified as triage classification 2 or 3. Exclusion criteria are as follows: incomplete patient records and patients who chose hospice care. Sixty-six patients completed the questionnaires. Multivariate regression was used to evaluate the determinants of overall satisfaction scores of the bidirectional referral. The two overall satisfaction scores of patients were high (> 7). Three independent variables, (1) overall satisfaction scores of medical care at Taipei City Hospital, Zhongxiao campus, (2) waiting times for examination, treatment, and test, and (3) a positive question regarding quality improvement of delivered care for patients and family explained 69.3% adjusted variability of the overall satisfaction scores of bidirectional referrals. Therefore, the policy of bidirectional referrals and direct admission into the wards of Taipei City Hospital, Zhongxiao campus, from the emergency department of National Taiwan University Hospital met the criteria for patient satisfaction and public accountability.

## Introduction

The Department of Health approved a health care project and planned the construction of a medical network project on April 26, 1975 in Taiwan. It helped integrate existing medical institutions and establish grading medical systems and referral systems, which promoted localized bidirectional referral. Furthermore, it adopted an integrated health care model to change patients’ medical behavior and prioritize medical care resources. The public's acceptance of bidirectional referrals has become the focus of many health organizations. The Department of Health also implemented the “Healthy Care for All” program in January 1991, which focused on patient-centered health care. The bidirectional referral program between National Taiwan University Hospital and Taipei City Hospital was launched on January 5, 2015. It promotes bidirectional communication and cooperation between medical centers and community hospitals and establishes standardized referral procedures to serve patients in the emergency department.

The bidirectional referral project between National Taiwan University Hospital and Taipei City Hospital can solve the emergent department crowding and decrease the length of emergency department boarding which may decrease work burden on emergent department staff, therefore emergent and urgent patients can be managed timely and adequately, resulting in decreased injury and illness severity. Afterwards, the outcomes (length of hospitalization and hospitalization mortality) of inpatients and referral patients are good^1–5^.

Studies on patient satisfaction and perceptions have recently attracted attention in primary care^6^ and modern patient-centered health care^7^, but very little information is available on the questionnaires that assessed the patients’ satisfaction with the bidirectional referral between a community hospital and medical center. This retrospective study analyzes effective questionnaires for referral patients between National Taiwan University Hospital and Taipei City Hospital, Zhongxiao campus, to evaluate the patients’ satisfaction with the referral system.

## Materials and methods

### Participants

The research subjects, referred from the emergency department of National Taiwan University Hospital to Taipei City Hospital, Zhongxiao campus, were recruited from April 2015 to December 2017. The ESI (Emergency Severity Index) is a 5-point emergency department triage classification that are used to classify patients into 5 types from I (most urgent) to V (least urgent) based on predicted acuity and resource requirements. There are 4 branch points (A-D) to guide triage tree. The ESI triage algorithm is as follows^8^: ESI 1—If patients require immediate lifesaving intervention (branch point A), they are classified as ESI 1.ESI 2—If patients do not require immediate lifesaving intervention (branch point A), but in high-risk situation, or confused/lethargic/ disoriented or severe pain/distress(branch point B), abnormal vital signs (branch point D), such as abnormal age-based heart rate and respiratory rate or oxygen saturation < 92% and needing many resource types(branch point C), they are classified as ESI 2. ESI 3—If patients require many resource types without abnormal vital signs (abnormal age-based heart rate and respiratory rate or oxygen saturation < 92%), without high-risk situation, or confused/lethargic/ disoriented or severe pain/distress and without needs of immediate lifesaving intervention, they are classified as ESI 3.ESI 4—If patients require one resource type without abnormal vital signs (abnormal age-based heart rate and respiratory rate or oxygen saturation < 92%), without high-risk situation, or confused/lethargic/ disoriented or severe pain/distress and without needs of immediate lifesaving intervention, they are classified as ESI 4. ESI 5.—If patients require none resource type without abnormal vital signs (abnormal age-based heart rate and respiratory rate or oxygen saturation < 92%), without high-risk situation, or confused/lethargic/ disoriented or severe pain/distress and without needs of immediate lifesaving intervention, they are classified as ESI 5. The selection criteria of the bidirectional referral service were emergency diagnosis of classification 2 and 3. Exclusion criteria included incomplete patient records and patients who chose hospice care. Patient satisfaction questionnaire surveys for bidirectional referrals between National Taiwan University Hospital and Taipei City Hospital, Zhongxiao campus, formed the database. From this, we extracted 66 satisfaction questionnaires. Informed consent was waived by Taipei City Hospital Research Ethics Committee.

### Questionnaires

The questionnaire was validated previously with two previous literatures, Taipei City Medical Journal 2018; 15(2):059–069 and Taipei City Medical Journal 2017;14(2):171–177, using the same questionnaire. All methods were performed in accordance with the relevant guidelines and regulations. All relevant patient satisfaction questionnaire materials will be stored in the office of the internal medicine department. We used the structural satisfaction questionnaire developed by Taipei City Hospital as the measurement tool; the satisfaction questionnaire was filled out on the day of discharge. Regarding the routine quality control survey of Taipei City Hospital, all relevant information of the self-administered structured questionnaire will be kept properly. Further, it is ensured that the patient's personal information is never revealed.

The questionnaires of this retrospective study included the following variables: age, gender, education and address of patients (county and city), patient perceptions of division, whether it was the patient’s first visit, hospitalization days, questions on medical divisions of delivered health care, overall satisfaction score of Taipei City Hospital, Zhongxiao campus, an open question on medical care at Zhongxiao campus, over satisfaction score of bidirectional referrals between National Taiwan University Hospital and Taipei City Hospital, Zhongxiao campus, and an open question on transferal from the National Taiwan University Hospital medical center to the Zhongxiao campus. Overall satisfaction scores of medical cares at the Zhongxiao campus and the overall satisfaction scores of the transferal from the National Taiwan University Hospital medical center to the Zhongxiao campus were rated on a 11-point scale. A 6-point Likert scale was used in the survey items of different dimensions of delivered health care: 1 = strongly disagree, 2 = disagree, 3 = acceptable, 4 = agree, 5 = strongly agree, 0 = no opinion.

The questionnaire included 64 survey questions with 2 open project questions.” The survey questions include three parts, Part1 with 7 testlets (“Environment”, “Administrative Efficiency “Service Attitude”, “Process”, “Patient Safety”, “Overall Evaluation of satisfaction of medical service of Zhongxiao campus” and “bidirectional referral”), Part 2 with information of backgrounds of hospitalization and Part 3 with information of basic data of patients or caregivers. In details, part I of the questionnaire was the satisfaction survey questions on the seven dimensions of delivered care under bidirectional care, which is rated on a 6-point Likert scale. It includes the evaluation of ward conditions and facilities, administrative efficiency, courtesy of staff service, medical service process, hospital-patient safety measures services, medical care at the relevant campus of Taipei City hospital, and satisfaction with transferal from the medical center to the relevant campus of Taipei City Hospital. Part II was the patient’s perception of the medical division. Part III A consisted of basic information of patients: gender, age, places of residence, highest education level, and hospitalization days. Part III B comprised basic information of the caregivers including the relationship of caregivers, gender, age, and highest education level. Medical malpractices were not referred to in the items of questionnaires dealing with risk management. The questionnaire surveys were conducted based on the principles of anonymity and confidentiality^9^.

### Statistical analysis

In the data analysis, the patient's identity was represented by a code. One-way ANOVA, Spearman correlation, and paired t-test were used according to the characteristics of variables^10^. The goal of the primary analysis was to choose significant items. Independent sample T-tests was used to test on mean of each item and each domain under different ESI groups. Missing data were treated with pairwise or listwise deletion in order to avoid bias^11^. Multivariate regression was used to analyze the determinants of the overall satisfaction scores of transferals from the medical center to Zhongxiao hospital. Age was included in the model due to the concept of geriatrics assessment guided health care^12^. The method of variable selection for entering the model is stepwise. SPSS version 15 was used for data management and analysis. The significance level was set at *P* < 0.05.

### Ethics declaration

Taipei City Hospital Research Ethics Committee with approval number TCHIRB-10705105E and TCHIRB-11009006E approved the study.

## Results

**Figure 1:**
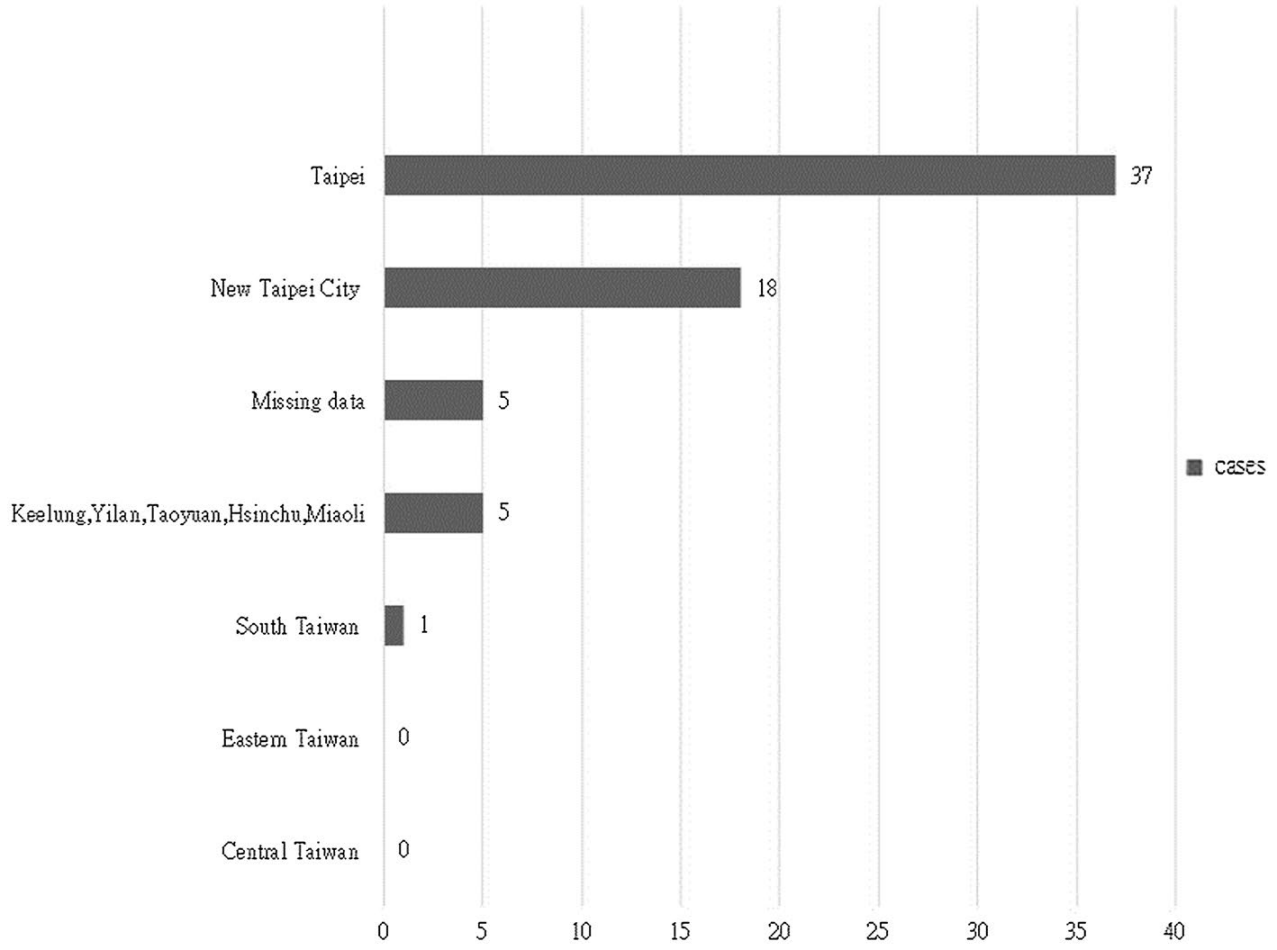
Places of residence of questionnaires’ respondents.

The descriptive data of domains of questionnaires are demonstrated in Table 1. The basic characteristics of patients are shown in Table 2. The mean age of the patients was 59.89 ± 18.29, and the male to female ratio was 1:1. The majority of patients were admitted to the division of Gastroenterology, Infection, and Chest. The highest level of education of the majority of respondents was junior high school, senior high school, and vocational school and college. Most respondents lived in Taipei city and New Taipei City (Fig. 1). All patients and representatives who were transferred from the emergency department of National Taiwan University Hospital to the Zhongxiao campus of Taipei City Hospital completed the questionnaires prior to their discharge. The questionnaire included 61 survey questions with 2 open project questions. The mean value of overall satisfaction scores of medical cares at Zhongxiao campus was 8.66. The mean value of overall satisfaction scores of bidirectional referrals was 9.00. Only the average of satisfaction scores of the administrative efficiency testlet has a significant difference between Emergency Severity Index 2 and 3. The average of satisfaction scores of Administrative Efficiency of the emergent (ESI = 2) patient (mean = 3.792) is less than that of the urgent (ESI = 3) patient (mean = 4.3). Because of limited number of responders, inclusion of the year in the analysis do not reach significant results.

**Table 1:** Descriptive data of domains of questionnaires.

Testlets	Mean	Standard deviation	Range	Missing (%)
Average of environment (9 items)	4.153	0.699	2–5	0.338
Average of administrative efficiency (6 items)	4.177	0.820	2–5	0
Average of service attitude (9 items)	4.325	0.763	1.88–5	1.180
Average of process (11 items)	4.425	0.599	2.18–5	0.276
Average of patient safety (5 items)	3.977	1.002	1.80–5	3.032
Average of overall satisfaction of hospital (5 items)	5.182	0.852	2.20–6	0.304
Average of overall evaluation of bidirectional referral (5 items)	5.533	0.904	3.4–10	2.424

**Table 2:** Profiles of questionnaires’ respondents (N = 66).

Variable	Number
Gender(total)	66
Female	33
Male	33
Age in years	59.89 ± 18.29
Highest levels of education(total)	66
Illiterate and elementary school	8
Junior high school	17
Senior high school and vocational school	15
Junior college	5
College	15
Post-graduate	5
Missing data	1
Patient perception of division	
Gastroenterology	13
Infection	13
Chest medicine	11
Nephrology	8
Neurology	5
General internal medicine	5
Others	3
Cardiovascular	2
Plastic surgery	2
Family medicine	1
Oncology	1
Endocrinology	1
General surgery	1
Places of residence(total)	66
Double Taipei city	55
Not double Taipei city	11

We simultaneously entered all significant independent variables into the regression model to explore the specific and clear predictors of respondents’ overall judgments on the quality of delivered care in the bidirectional referral. Three independent variables, waiting times for examination, treatment, and test (standardized beta coefficient = 0.386), overall satisfaction scores of medical care at the Zhongxiao campus (standardized beta coefficient = 0.321), and the positive question on quality improvement of delivered care for patients and family (standardized beta coefficient = 0.363), explained 69.3% adjusted variability of the overall satisfaction scores of bidirectional referrals (Table 3 and 4).The results of collinearity diagnosis (VIF and tolerance)were negative and shown in the Tables 3 and 4.

**Table 3:** Results from multivariate regression optimization model while using listwise deletion method for missing data).

	Regression parameters					
Predictors	Beta coefficients	Standard error of estimate	Standardized beta coefficients	*P* value	Collinearity statistics	
					tolerance	VIF
Constant	-0.133	0.083		0.113		
Over satisfaction of this hospital (0–10)	0.343	0.119	0.321	0.006	0.473	2.112
Improve the quality of medical treatment for patients and their families (0–5)	0.473	0.108	0.363	< 0.001	0.859	1.165
Reasonable waiting time for the results of examinations, check-up, treatment, operation report (0–5)	0.530	0.146	0.386	0.001	0.518	1.930
F value = 40.170, *P* < 0.001						
Adjusted R Square (R^2^) = 0.693

**Table 4:** Results from multivariate regression optimization model (pairwise deletion method for missing data).

	Regression parameters					
Predictors	Beta coefficients	Standard error of estimate	Standardized beta coefficients	*P* value	Collinearity statistics	
					tolerance	VIF
Constant	1.416	0.717		0.054		
Over satisfaction of this hospital (0–10)	0.274	0.095	0.321	0.006	0.473	2.112
Improve the quality of medical treatment for patients and their families (0–5)	0.604	0.138	0.363	< 0.001	0.859	1.165
Reasonable waiting time for the results of examinations, check-up, treatment, operation report (0–5)	0.570	0.158	0.386	0.001	0.518	1.930
F value = 40.170, *P* < 0.001						
Adjusted R Square (R^2^) = 0.693						

Analysis of residuals revealed that the linear regressions meet the following linear assumptions.1. Independence and no auto-correlation of the errors: In case of treating missing data with pairwise deletion or listwise deletion and according to statistical table of Durbin-Watson test, when α = 0.05, the critical value is less than 1.421 or greater than 1.674 when the number of independent variables are 3 and the sample size is 50, and the critical value is less than 1.452 or greater than 1.681 when the sample size is 55. With 3 independent variables and 53 samples, the calculated Durbin-Watson value = 1.754, which is greater than the upper bound of the two critical values mentioned above. Using R (programming language) to assist the calculation, the p-value is 0.372 is greater than 0.05, which means that the residuals of the regression model are independent of each other. 2: Homoscedasticity: From the scatter plot of standardized residuals and predicted values, it can be seen that the data are not evenly scattered up and down along the horizontal line of 0. Use R language for test of constant variance assumption, the variance of the residuals of the regression model is homoscedastic. 3: Normality of the error distribution: We first took logarithmic transformation of the original value of  the selected independent variables. According to the Shapiro–Wilk normality test, the significance = 0.125 > α = 0.05, so the residuals meet the normality assumption.4. No or little multicollinearity: The values of VIF are all less than 10, which means that the selected variables have no collinearity problem. The reliability and validity of the measurement tool have been described in Table 5.

**Table 5:** Comparison of the reliability of questionnaires, the validity of questionnaires and the explanatory power of models between listwise deletion method and pairwise deletion method while treating missing data.

	Listwise deletion	Pairwise deletion
Environment Cronbach’s α	0.894 (n = 64)	0.894 (n = 64)
Administrative efficiency Cronbach’s α	0.898 (n = 66)	0.898 (n = 66)
Service attitude Cronbach’s α	0.875 (n = 62)	0.875 (n = 62)
Process Cronbach’s α	0.929 (n = 65)	0.929 (n = 65)
Patient safety Cronbach’s α	0.746 (n = 61)	0.746 (n = 61)
Overall evaluation, Cronbach’s α	0.863 (n = 65)	0.863 (n = 65)
Referral Cronbach’s α	0.888 (n = 64)	0.888 (n = 64)
Explainable percentage of variation (Validity Analysis)	84.536% (n = 53)	95.986% (n = 66)

The reliabilities of questionnaires of listwise and pairwise deletion methods are equal. The validity of questionnaires and the explanatory power of model pairwise deletion method is better than that of listwise method.

## Discussion

The main findings of this study were as follows: the mean values of the overall satisfaction scores of medical care at the Zhongxiao campus (8.66) and bidirectional referral (9.00) were both above 0.70^13^; these are comparable with those reported in large scale researches^14^. Only the average of satisfaction scores of the administrative efficiency domain has a significant difference. The average of the emergent (ESI = 2) patient (mean = 3.792) is less than that of the urgent (ESI = 3) patient (mean = 4.3).Three independent variables, overall satisfaction scores of medical care at the Zhongxiao campus, waiting times for examination, treatment, and test, and the positive question on quality improvement of delivered care for patients and family, were the determinants that explained 69.3% variability (adjusted R square) of the overall satisfaction scores of bidirectional referrals.

The operational definitions of the three independent variables are as follows: waiting time is defined as the total time a patient spends in a facility from arrival at the registration desk until the time she/he leaves the facility or last service.

An overall satisfaction score is a measurement of overall satisfaction level through a one-question survey. It asks users to rate their experience with the services of hospital. Overalls answer based on a scale from “extremely dissatisfied (0)” to “extremely satisfied (10)”. Health organization can use the score to determine their satisfaction levels at crucial touchpoints like the overall evaluation with environment, administrative efficiency, service attitude, process, patient safety. Positive question on quality improvement of delivered care for patients and families means that the bidirectional referral can positively influence the quality improvement of delivered care for patients and families.

The cycle of plan-do-study-act (PDSA) can be used as the improvement guide and approach to enhancing the performance of health care organizations, using questionnaires as one kind of learning data with comprehensively statistical analyses to support improvement efforts^15^. Based on the theory of Nolan’s model, the cycle of plan-do-study-act (PDSA) can be used as a basis for the improvement of the bidirectional referral system between National Taiwan University Hospital and Taipei City Hospital^16^. In addition to traditional key performance indicators, such as morbidity and mortality^17^ and rate of transfer back to National Taiwan University Hospital, analyzing questionnaires dealing with patient satisfaction has become an important tool of evaluation in a specific domain of patient -centered care, leading to an improvement of the bidirectional referral system between National Taiwan University Hospital and Taipei City Hospital^18^. These questionnaires are multidimensional modalities and surveys that evaluate variable aspects of the delivery of health care.

There are important objective aspects of self-measuring metrics of the quality of the six dimensions (access to services, relevance to need, effectiveness for individual patients, equity, social acceptability and efficiency, and economy) of medical service^19^. Modalities of the evaluation of patient satisfaction include an interview in a hospital, telephone interview after discharge, traditional mails, e-mails, computer questionnaires^20^, and document questionnaires^17^. Different aspects of patient satisfaction are as follows: timeliness^10,14,21^, humaneness, cost^22^, patient-centered and shared decision making and shared informativeness^18,21,23^, facilities, overall quality, outcome and quality of life^20^, competence^24^, continuity^21,22^, courtesy and communication skills of health care providers^23,24^, bureaucracy, attention to a psychological problem^14,18^, cleanliness and hospital environment^17,24^ and access^9^.

Although patient expectations and perceptions may be influenced by culture, characteristics of the patient^25^, criteria of quality of patient^7^, previous experience and needs of patients, the concept of patient-centered care^26^, and patient partnership strategy^27^ equal patients as different medical knowledge experts and treat patients as partners. A systemic survey of patients’ perceptions, the expectation is important to improve the quality of modern health care of medical knowledge grown-up patients and their family.

Interventions to improve patient satisfaction may be important and valuable because there is evidence that satisfied patients are more likely to adhere to their health care routine. This, in turn, is related to effective treatment-related clinical outcomes^14^. Higher patient satisfaction means fewer demands for emergent department medical services^28^. From the perspective of health care organizations, one of the chief reasons for surveying patient satisfaction is to find targeted information to improve delivered care under bidirectional referral processes in specific service areas. This is important because of the unpredictable nature of problems encountered in such referral programs.

Modifications must be made to achieve improvement in the determinants with a largely standardized beta coefficient in the optimization mode of overall satisfaction^24^ among the vulnerable groups, which can represent the primary opinions of the referral patients from National Taiwan University Hospital to Taipei City Hospital, Zhongxiao campus. The average of overall satisfaction of Administrative Efficiency of the emergent (ESI = 2) patient (mean = 3.792) is less than that of the urgent (ESI = 3) patient (mean = 4.3). But, the results of determinants of multivariate regressions showed that the positive beta coefficient of waiting times for tests, examinations, and treatments represented the majority of the patients’ opinions in this referral program. Waiting times influence the perception of safety and confidence in the medical care system, which may change patients’ attitudes toward medical advice. From a management point of view, our findings may help healthcare providers organize limited resources more successfully in the patient satisfaction refinement program under the bidirectional referral between National Taiwan University Hospital and Taipei City Hospital, Zhongxiao campus. The traffic flow and each node of test, examination, and treatment of patients should be examined for improvement and modification.

Other types of waiting times, such as time spent in the waiting room, time with health providers, and consultation time with physicians, were not considered as the determinants of overall satisfaction of bidirectional referral in this study. Patient advisors can aid patients to complete survey questionnaires and thus avoid missing data^17^. The short duration between discharge and completion of survey questionnaires also decreases the recall bias. Other forms of satisfaction surveys, such as regular mail and telephone calls after discharge have the limitation of recall bias. Further, a hundred percent response rate after the administration of the questionnaires in this study eliminated selection bias. A previous study showed that waiting times more than 30 min may provoke low satisfaction^14^.

Additionally, in response to the open question, one respondent praised the medical personnel and nursing intern for their attitude. Therefore, screening items for specific domains referred to in open questions may improve the design of future questionnaires ^29,30^.

## Conclusions

The results of the study show that the bidirectional referral policy acquired good overall satisfaction. The determinants of overall satisfaction are composed of three positive responses. The negative response empowers policy and health care managers and providers to prioritize improvement initiatives if it is related to outcome^31^, such as hospitalization day. With information and an understanding of the determinants of questionnaires, we can ensure that health care is patient-centered and value-based. Systemic and regular (3 months, 6 months, or 1 year) surveys of patient satisfaction metrics is a strong tool for organizing and improving targeted resources and health care delivery^28^. More specific and clear results will need to be accumulated in the future analysis of questionnaires. We hope to continue our efforts in this direction and review the analysis of specific and clear findings to improve the quality of the bidirectional referral system of patient care. Simultaneously, we can extend this model to other medical institutions. Although the adjusted explanatory power (adjusted R^2^) of our questionnaire model using pairwise deletion method and listwise deletion for treating missing data both are 0.693, we have acknowledged a potential future study to overcome the limitation of small sample size.

## Data Availability

The raw data that support the findings of this research are available from the office of internal medicine and the corresponding author (Jenn Yeu Wang, MD) but restrictions apply to the availability of these data, which were used under permissions for the current study, and so are not publicly available. The raw data were already de-identified before analysis. De-identified Data are however available from the corresponding author upon reasonable request and with permission of institutional review board.
